# Neurophysiological Correlates of Trait Impulsivity in Parkinson’s Disease

**DOI:** 10.1002/mds.28625

**Published:** 2021-05-13

**Authors:** Lucia Ricciardi, Petra Fischer, Abteen Mostofi, Gerd Tinkhauser, Flavie Torrecillos, Fahd Baig, Mark J. Edwards, Erlick A.C. Pereira, Francesca Morgante, Peter Brown

**Affiliations:** 1 Neurosciences Research Centre, Molecular and Clinical Sciences Research Institute, St George’s University of London, London, United Kingdom; 2 Medical Research Council Brain Network Dynamics Unit, Nuffield Department of Clinical Neurosciences, Oxford, United Kingdom; 3 Department of Neurology, Bern University Hospital and University of Bern, Bern, Switzerland; 4 Department of Clinical and Experimental Medicine, University of Messina, Messina, Italy

**Keywords:** Parkinson’s disease, impulsivity, impulsive compulsive behavior disorders, deep brain stimulation, subthalamic nucleus

## Abstract

**Background:**

Impulsivity is common in people with Parkinson’s disease (PD), with many developing impulsive compulsive behavior disorders (ICB). Its pathophysiological basis remains unclear.

**Objectives:**

We aimed to investigate local field potential (LFP) markers of trait impulsivity in PD and their relationship to ICB.

**Methods:**

We recorded subthalamic nucleus (STN) LFPs in 23 PD patients undergoing deep brain stimulation implantation. Presence and severity of ICB were assessed by clinical interview and the Questionnaire for Impulsive-Compulsive Disorders in PD-Rating Scale (QUIP-RS), whereas trait impulsivity was estimated with the Barratt Impulsivity Scale (BIS-11). Recordings were obtained during the *off* dopaminergic states and the power spectrum of the subthalamic activity was analyzed using Fourier transform-based techniques. Assessment of each electrode contact localization was done to determine the topography of the oscillatory activity recorded.

**Results:**

Patients with (n = 6) and without (n = 17) ICB had similar LFP spectra. A multiple regression model including QUIP-RS, BIS-11, and Unified PD Rating Scale-III scores as regressors showed a significant positive correlation between 8–13 Hz power and BIS-11 score. The correlation was mainly driven by the motor factor of the BIS-11, and was irrespective of the presence or absence of active ICB. Electrode contact pairs with the highest α power, which also correlated most strongly with BIS-11, tended to be more ventral than contact pairs with the highest beta power, which localize to the dorsolateral motor STN.

**Conclusions:**

Our data suggest a link between α power and trait impulsivity in PD, irrespective of the presence and severity of ICB.

Cognitive and neuropsychiatric disorders are common in people with Parkinson’s disease (PD) and have a major impact on patients’ and caregivers’ quality of life. The high prevalence of cognitive and neuropsychiatric disorders has led to the suggestion that PD should more accurately be conceptualized as a neuropsychiatric disorder.^1^

Among the neuropsychiatric symptoms of PD, impulsive compulsive behaviors (ICB) represent a frequent source of disability with a 5-year cumulative incidence of 46.1%.^2^ ICB comprise impulse control disorders such as pathological gambling, compulsive shopping, hypersexual behavior, and compulsive eating, and related disorders such as punding/hobbyism and dopamine dysregulation syndrome. Interplay between exposure to dopaminergic medications and endogenous characteristics in people with PD seem to underlie the generation of these behavioral disorders.^3^ In particular, certain personality traits have been suggested to predispose people with PD to develop ICB, namely a novelty seeking profile and high level of impulsivity.^4,5^ Impulsivity has been conceptualized as a multifactorial construct accounting for at least three dimensions: trait impulsiveness, impulsive action, and impulsive choices.^6^

Clinical and experimental evidence suggests that the subthalamic nucleus (STN) plays a key role in the inhibitory control of both action and behavior.^7^ The STN is also the main target of deep brain stimulation (DBS), a well-recognized effective treatment for people with advanced PD. STN-DBS can provide the opportunity to record STN local field potentials (LFPs) and such recordings have provided important information on the pathophysiology of motor features of PD and the effects of treatment with levodopa. However, relatively little is known about the LFP correlates of non-motor symptoms in PD and in particular ICB. An influential resting state study reported greater theta band activity in the ventral STN in PD with active ICB in the “*on* medication” condition,^8^ but the extent to which this might reflect trait differences as opposed to a particular neural circuit response to levodopa medication remains unclear. Indeed, to date, no studies have investigated the electrophysiological markers of trait impulsivity, which might predispose patients to ICB. Accordingly, here, we evaluate the LFP correlates of trait impulsivity in a cohort of PD patients with and without ICB, assessing trait impulsivity with the Barratt Impulsivity Scale (BIS-11), a validated and widely used scale.

## Methods

### Patients and Clinical Assessments

Consecutive PD patients undergoing bilateral STN-DBS who gave their written informed consent to participate in the experiment (IRAS project ID: 75154) were recruited at St. George’s University Hospital, London, United Kingdom (UK).

Motor symptoms were assessed before DBS (205 + 61 days before) and included a levodopa challenge test as per Core Assessment Program for Surgical Interventional Therapies in PD (CAPSIT-PD) protocol with rating of Unified Parkinson’s Disease Rating Scale part III (UPDRS-III) in the practically defined *off* and *on* medication state.^9^ Presence and severity of dyskinesia were evaluated with the UPDRS-IV (total score and sum of sub-items 32–35 for dyskinesia) and the Rush Dyskinesia Rating Scale (RDRS). The diagnosis of ICB relied on a semi-structured interview based on ICB diagnostic criteria,^10^ which was performed on the same day as the levodopa challenge test and confirmed at the time of the study. ICB was defined as “Active” if it was ongoing at the time of study entry. “Past” ICB were defined as patients who had ICB at any point during their disease, but were in remission at the time of the study. The Questionnaire for Impulsive-Compulsive Disorders in PD-Rating Scale (QUIP-RS)^11^ and BIS-11^12^ were used to measure severity of ICB and trait impulsivity, respectively. The BIS-11 scale is a 30-item questionnaire measuring trait impulsivity, comprising the following factors: attentional (attention and cognitive instability), motor (motor and perseverance), and non-planning (self-control and cognitive complexity). Presence and severity of depression was evaluated with the Hamilton depression rating scale (HDRS).

### Neurophysiological Testing

DBS implantation was a staged procedure. LFP recordings were made between 3 and 5 days postoperatively, while electrode leads were still externalized and before implantation of the subcutaneous pulse generator. Patients were recorded OFF medication.

LFP recordings were made for a minimum of 3 minutes (maximum 10 minutes) while patients were seated comfortably at rest in an armchair with their eyes open. Signals were recorded with a TMSi-Porti amplifier (TMS International, Oldenzaal, the Netherlands). The ground electrode was placed on a forearm. LFP signals were sampled at 2048 Hz and common average referenced. LFPs from quadrapolar electrodes were offline reconfigured to give a bipolar contact arrangement between the four levels so that each electrode afforded three bipolar signals on the left (from bottom to top: L01, L12, L23) and right (R01, R12, R23). In 17 patients, directional leads (15 Boston Scientific and 2 Abbott Neuromodulation) were implanted and directional contacts of one level were connected together to form one “ring” contact, and then bipolar contacts were constructed offline as before. Note that the contributions of the three segmented contacts to the ring contact average may not be equal considering that impedances may have differed between the segmented contacts, although the latter have similar dimensions.

In one patient (case 16) the lead was a non-directional octopolar one and we selected the four most ventral electrodes per side, after checking that they were located adjacent to or in the STN. In one patient (case 7) we excluded one channel (R23) because of the presence of artifacts. Bipolar montages between adjacent contact pairs were used because they limit the effects of volume conduction from distant sources.^13^

The data were first visually inspected using Spike2 Software (CED Cambridge Electronic design limited, UK). Sections contaminated by artifacts (eg, signal saturation or movement artifact) were removed from the data set. The average duration of analyzed artifact-free data was 262 ± 161 seconds. For the main analysis LFP activity in the STN region was computed by averaging across all bipolar channels of each electrode to avoid any channel selection bias. Spectral analysis based on the fast Fourier transform was performed in Spike2. Non-overlapping windows of 2048 data points were analyzed, affording a spectral resolution of 1 Hz. LFP power at frequencies ≤4 Hz was excluded from further analysis, because this is subject to movement artifacts and heavily influenced by the 1/f nature of the signal rather than by oscillations. We considered power between 5–35 Hz as our range of interest because it includes the θ-α band previously implicated in ICB^8^ and the β band associated with motor impairment, but also implicated in response inhibition in some studies. Power in this range of interest was normalized by dividing it by the sum of the power over 5–395 Hz (excluding line noise peaks at 50 ± 5 Hz and its harmonics) and multiplying by 100 to give a percentage. This step is necessary to limit the variation in spectral power between sides and subjects because of variance in targeting and impedance.

Additional information is in the Supporting Data.

### Statistical Analysis

In our core analysis, we used a general linear model (GLM) to investigate if STN power averaged across all contacts and the left and right hemispheres was significantly related to ICB severity (QUIP-RS) and trait impulsivity (BIS-11). To assess if motor symptoms accounted for variations in the LFP we also added the UPDRS-III scores to the GLM. The predictor variables were z-transformed to enable a better comparison of the regression coefficients. The model was fitted using the MATLAB function *fitlm* with a bisquare weighting function to compute a robust fit (v. 2019b, The MathWorks, Natick, MA). Regression coefficients were computed separately for frequencies ranging from 5 to 35 Hz and corrected for multiple comparisons with a cluster-based permutation procedure described in the Supporting Data.

In subsequent exploratory analyses we took the average power over the 8–13 Hz frequency range found to be significant using the above approach and determined its relationship with the three components of the BIS-11 captured by the attentional, motor and non-planning sub-scores, and with the HDRS score. Spearman’s correlation coefficients were computed to compare the size of correlations. STN power was averaged across the left and right hemispheres in each subject before the correlation analysis unless otherwise stated. Finally, we determined the relationship between α power and BIS-11 scores when extracting power from the bipolar contact configuration that had the highest α power and contrasted this with that from the contact pair with the highest β power.

## Results

We evaluated 23 PD patients undergoing STN-DBS (five women, mean age 59.0 ± 6.2 years, mean disease duration 9.8 ± 3.7; Table 1). According to the semistructured clinical interview, six patients had active ICB and 17 had no-active ICB. Demographic and clinical differences between these subgroups are summarized in Table 2. Within the no-active ICB group seven patients had ICB in the past, whereas 10 patients had never had any ICB.

### Dependencies between LFP Power and Symptom Complexes

**Fig. 1:**
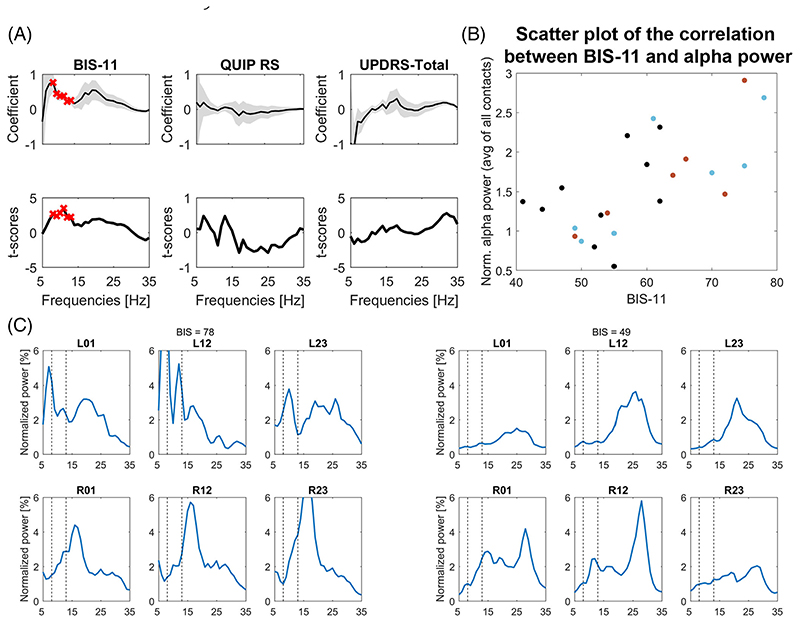
Multiple linear regression with STN power averaged across all bipolar signals as criterion and BIS-11, QUIP-RS, and UPDRS-III scores as predictors. (**A**) A significant cluster (see red crosses) was found between 8–13 Hz power for the BIS-11 scores, indicating a positive relationship between α power and the strength of impulsivity (n = 23). The t-scores for the UPDRS-III predictor showed a positive deflection in the high β range, but the effect did not survive correction for multiple comparisons. An exploratory correlation with just contralateral hemibody bradykinesia and rigidity items was significant over 31–32 Hz (inclusive): ρ = 0.44, *P* = 0.035. Gray shadow in upper panels shows the standard error of the coefficients. (**B**) Scatter plot of BIS-11 scores and 8–13 Hz α power averaged across all bipolar contacts. The three different colors of the points denote whether ICB were currently, previously or never present (red, active; blue, past; black, never). (**C**) Example STN LFP power spectra. Left-hand and right-hand sets of six panels come from patients with a high BIS-11 score of 78 and a low BIS-11 score of 49, respectively. Y-axes showing normalized power have been optimized to view the α band activity shown between the two vertical dashed lines. Each set of six panels shows the spectra recorded at the three bipolar contacts (01, 12, and 23) on the left (L) and right (R) sides. Contact 0 is the most ventral on each side. [Color figure can be viewed at wileyonlinelibrary.com]

**Fig. 2:**
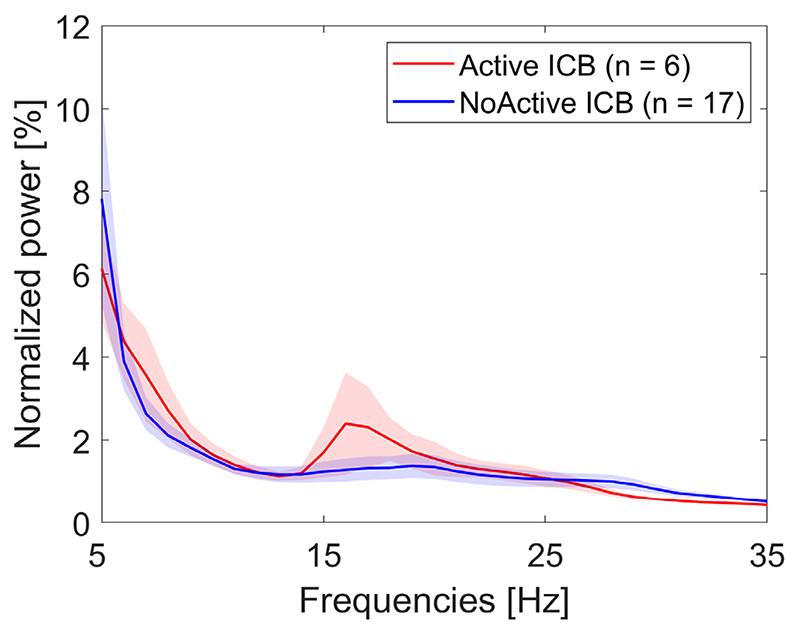
The power spectra of Active (n = 6) and NoActive (n = 17) ICB patients showed no significant differences. Statistics were computed using a non-parametric cluster-based permutation procedure. Shaded regions show standard errors. [Color figure can be viewed at wileyonlinelibrary.com]

To explore statistical dependencies between LFP power over the 5–35 Hz range and behavioral features, we computed a multiple regression model including the QUIP-RS score, BIS-11 score, and UPDRS-III score as predictors and LFP power as outcome variable. We found significant positive regression coefficients for LFP power estimates between 8–13 Hz and BIS-11 score after correcting for multiple comparisons over the whole 5–35 Hz frequency range with the cluster-based permutation procedure (Fig. 1A). Although the active ICB group had a power peak in the low β frequency range, which was not present in the no-active ICB group (Fig. 2), the regression analysis did not result in a significant cluster in this frequency band.

Neither the coefficients for the QUIP-RS or UPDRS-III score were significant in this model after correction for multiple comparisons, although those for the UPDRS-III did show a peak in the low β range.

This dependency between α power and BIS-11 score was confirmed in a model with power averaged across 8–13 Hz as outcome variable (*F*-statistic = 4.21, *P* = 0.019; BIS-11: t-statistic [t-stat] = 3.17, *P* = 0.005; QUIP-RS: t-stat = –0.07, *P* = 0.942; UPDRS: t-stat = –0.55, *P* = 0.589). Therefore there was a link between STN oscillatory activity in the α range and impulsivity, independent of motor symptoms and the severity of ICB. To visualize this relationship, we correlated 8–13 Hz power with the BIS-11 scores across patients (Spearman’s ρ = 0.67; Fig. 1B). To test if the diagnosis of active ICB played a role in influencing the correlation, we computed another regression model with “active ICB”/”no-active ICB” as categorical variable, age, and the total BIS-11 score as predictors. Again, only the coefficient for the BIS-11 scores was significant (BIS-11: t-stat = 2.81, *P* = 0.011; active/no-active ICB: t-stat = –0.59, *P* = 0.562, age: t-stat = 0.15, *P* = 0.884, interaction BIS-11*active/no-active: ICB t-stat = 0.65, *P* = 0.525).

The lack of significance of the active ICB diagnosis variable (t-stat = 0.587, *P* = 0.564) and lack of interaction (t-stat = 0.723, *P* = 0.5), together with the significant BIS-11 t-statistic (t-stat = 3.04, *P* = 0.007) highlights that the correlation between BIS-11 and α power was present irrespective of the presence of active ICB. The three PD subgroups (Never ICB, Past ICB, and Active ICB) are highlighted in red, blue, and black in Fig. 1B. It shows that a positive relationship was present within each subgroup (Never ICB: ρ = 0.48, *P* = 0.160 [n = 10]; Past ICB: ρ = 0.79, *P* = 0.048 [n = 7]; Active ICB: ρ = 0.83, *P* = 0.058 [n = 6]). The relationship was only significant in the Past ICB group, but the number of patients in each subgroup was small. Example spectra of two patients, one with a low and one with a high BIS score, are shown in Figure 1C.

Then we performed a series of exploratory correlations aimed at refining the link between α activity and the BIS-11 score. Impulsivity is thought to comprise three components dissociable as the attentional, motor, and non-planning second-order factors in the BIS-11. Accordingly, we correlated each of these BIS-11 sub-scores with the normalized LFP power averaged over the 8–13 Hz frequency bins. Alpha power correlated most strongly with the motor component sub-score (ρ = 0.61, *P* = 0.002, unadjusted and significant after Holm-Bonferroni correcting for multiple comparisons), followed by the attentional sub-score (ρ = 0.53, *P* = 0.010), although the latter was also correlated with the motor component sub-score (ρ = 0.67, *P* < 0.001, significant after multiple comparison correction). The non-planning component was the most weakly correlated with α power (ρ = 0.42, *P* = 0.047), and also did not correlate significantly with the motor subcomponent (ρ = 0.14, *P* = 0.518). Given the correlation between the motor and attentional subcomponents, we performed partial correlations between the BIS-11 motor sub-score and α power while controlling for attention (ρ = 0.39, *P* = 0.072) and, conversely, between the BIS-11 attention sub-score and α power, controlling for the motor sub-score (ρ = 0.19, *P* = 0.400).

We also performed partial correlations between α power and BIS-11 controlling for: depression, dopaminergic therapy (total levodopa equivalent daily doses [LEDD] and LEDD dopamine-agonists) and dyskinesia severity. The correlation remained high (HDRS: ρ = 0.70, *P* = 0.001, n = 21 instead of 23, because for two patients the depression scores were not recorded; total LEDD: ρ = 0.67, *P* = 0.001; LEDD dopamine-agonists: ρ = 0.67, *P* = 0.001; UPDRS-IV dyskinesia sub-scores: ρ = 0.65, *P* = 0.001; RDRS scores: ρ = 0.67, *P* = 0.001). Together these results suggest that the correlation between BIS-11 and α power is dominated by the correlation between the motor impulsivity component and α power.

### Source of the LFP Activity Correlating with Trait Impulsivity

We found no evidence of lateralization when correlating the BIS-11 score with the average normalized 8–13 Hz LFP power obtained from two subsets (left and right) of contacts obtained in the two hemispheres (Supporting data). Averaging these two subsets again confirmed the significant relationship between alpha power and BIS-11 scores (averaged between hemispheres [n = 23]: ρ = 0.61, *P* = 0.002).

**Fig. 3:**
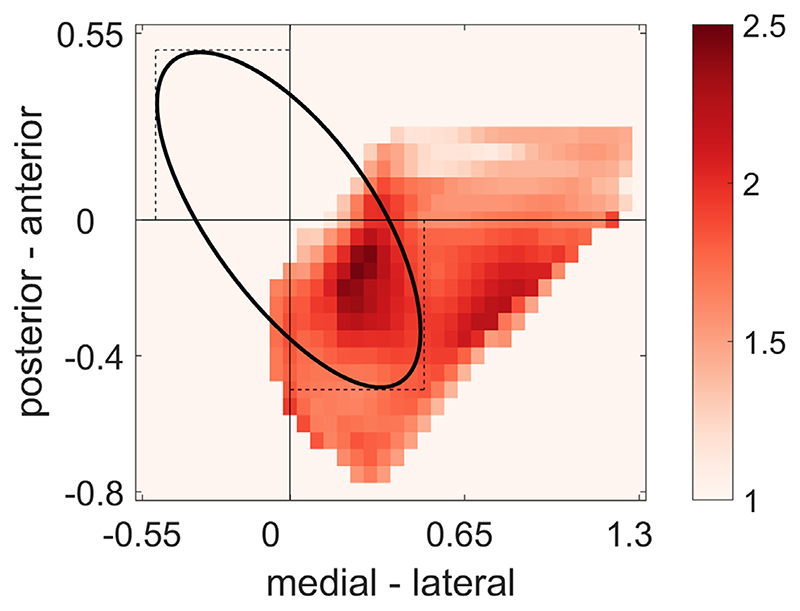
Distribution of α power. Power averaged across contact positions normalized relative to the STN poles (anterior pole: x = –0.5, y = 0.5; posterior pole: x = 0.5, y = –0.5), reducing the between-subjects variability because of differently sized subhalamic nuclei. Distances are relative to the STN centroid located at [0, 0], where the horizontal and vertical lines cross. The black ovoid represents the dimensions of the idealized STN. Note that contacts were positioned such that not all of the STN was covered. [Color figure can be viewed at wileyonlinelibrary.com]

Contacts were located predominantly in the posterolateral “motor STN,” as shown by the distribution of α power within the recorded region of the STN (Fig. 3). A hotspot is seen that is inset from the postero-lateral border of the STN by approximately a quarter of the distance between the nucleus’ poles. Another way of determining whether the α band signal correlating with the BIS-11 comes primarily from the motor STN or not, is to contrast the correlations established above with correlations based on α power from the bipolar contacts that had the highest β (rather than alpha) power per electrode. This is because the bipolar contact with the highest β power tends to localize to the dorsal posterolateral “motor” STN.^14^ Use of the highest β contact pair led to weakening of the relationship between α power and BIS-11 scores (averaged between hemispheres [n = 23]: ρ = 0.37, *P* = 0.080; compared to ρ = 0.61, *P* = 0.002 for peak α contacts). This suggested that the source of the α power that correlates best with BIS-11 scores may be slightly different to the β power hotspot in the motor region of the STN. Indeed, the relationship between the different sets of contact pairs supported a slightly more ventral source for the peak α activity that maximally correlates with the BIS-11 score. The bipolar contact with the highest α power (and the stronger α-BIS correlations) was ventral in 21 of 46 (46%) instances, overlapping in 17 of 46 (37%) instances and dorsal only in 8 of 46 (17%) instances when compared with the bipolar contact with the highest β power.

## Discussion

This is the first study in people with PD to explore STN LFP correlates of trait impulsivity per se. We recorded LFPs at rest in the “*off* medication” condition and found a positive correlation between the oscillatory activity in the α band (8–13 Hz) and the degree of trait impulsivity in PD patients, irrespective of the presence and severity of active ICB. STN LFP power spectra showed no significant differences between patients with and without active ICB.

### Trait Impulsivity Is Separate from ICB

The relationship between impulsivity and ICB is complex and still not very clear. Indeed, impulsivity is common in PD, even in the absence of ICB. It is a psychological construct characterized by poor control of thoughts and actions with a tendency to rapidly respond to impulses and environmental cues despite potential negative consequences. Therefore impulsivity itself has obvious aspects in common with ICB, and indeed previous studies have shown high trait impulsivity (ie, a high score on the Barrat-11 scale) in PD patients with ICB. However, there is no evidence on whether this is a specific component of ICB or a predisposing factor that might be present in a number of these patients.^4,15^ Moreover, clinical studies looking at differences in impulsivity between PD patients with and without ICB have reported inconsistent results with higher^4,15,16^ or comparable levels of trait impulsivity measured by BIS-11.^17^ Behavioral aspects of impulsivity, such as action and choice impulsivity have also been studied in relation to ICB, again with inconsistent findings. Indeed, on behavioral testing, the presence of ICB in PD have been associated with increased impulsivity,^5,18,19^ less impulsivity,^20,21^ or no difference compared to PD without ICB.^22,23^ Our data expand this previous literature and suggest that ICB, encompassing compulsive components, do not necessarily share the same alterations of neural activity as underlie trait impulsivity. Hence, patients may exhibit the two behavioral phenomena to different degrees, and trait impulsivity can be present even in the absence ofICB. Finally, the demonstration of a physio-marker for trait impulsivity described here would provide the opportunity to assess its modulation by short and long-acting dopaminergic medications and correlation to ICB change using novel sensing DBS devices.

### Relationship to Other LFP Studies

Previous LFP studies in PD patients have reported a modulation of low frequency activity in the α-θ band during tasks assessing inhibitory control of action as a proxy of impulsivity, such as the stop-signal, Stroop and Flanker tasks.^24^ These results support the role of STN and low frequencies in modulating action and choice inhibition in PD. However, whether impairments in these tasks are relevant in the generation of behavioral and affective symptomatology is unclear.

Only two studies have specifically carried out LFP recordings in the STN in PD patients focusing on ICB. Rodriguez-Oroz et al^8^ reported θ activity in the ventral region of STN in ICB in the *on* medication condition. In addition, Rosa et al^25^ demonstrated that PD patients with pathological gambling, a specific type of ICB, adopted a risky strategy during decision making and showed a greater change in low-frequency power in the STN when evaluating trials with conflict compared to those without. Like Rodriguez-Oroz et al,^8^ we found no difference between PD with and without ICB in LFP patterns recorded in the “*off* medication” condition, suggesting perhaps that the differences they identified *on* medication might reflect a specific neural circuit response to dopaminergic stimulation in ICB.

Impulsivity is a multi-faceted construct and this multi-dimensionality is reflected in the BIS-11 factor structure. Our data suggest that the positive correlation between α power and BIS-11 scores was mostly driven by the second order motor factor rather than by the non-planning or attentional factors of the BIS-11. Both mapping of the α power distribution in space across subjects and along the electrode within subjects raised the possibility that α power correlating with the BIS-11 tends to arise from an area slightly ventral to the dorsolateral motor area. This is consistent with higher resolution microelectrode recordings, which identify a region more ventral to the motor area that is characterized by 7–10 Hz activity.^26^

The dominant correlation between α power and the BIS-11 motor factor is also consistent with a recent structural connectivity study that associates impulsivity in PD to STN connectivity, with the response inhibition or “stopping” network linking left supplementary area and right inferior frontal gyrus.^27^ This network is thought to involve hyperdirect pathway input to the STN,^28^ with the strength of hyperdirect connections correlating with the efficacy of stopping.^29,30^

Impulsivity is a key component in a number of other neuropsychiatric disorders, and it is therefore interesting that, among these, elevated α band power at rest has been reported in the ventralis oralis complex of the thalamus of patients with Tourette’s syndrome,^31^ in the bed nucleus of stria terminalis or in subgenual cingulate area in patients with major depression,^32^ and in the nucleus accumbens in patients with addiction or substance use disorders.^33^ This raises the possibility that α activity might serve as a biomarker for behavioral impulsivity across neuropsychiatric disorders.

### Study Limitations

First, our sample included six patients with active ICB; hence, the absence of state-dependent differences in STN LFPs may have been a type 2 error, particularly because group differences might have been attenuated by a post-operative stun effect. Second, although our data demonstrate that α power and trait impulsivity are correlated, they cannot ascertain whether this reflects a primarily pathological or a secondary, possibly compensatory, process. Third, the precision of our neurosurgical targeting meant that we could not sample directly from the ventromedial limbic and associative parts of the STN. Fourth, previous LFP studies in PD have shown a correlation between the reduction in α power in the STN in response to emotionally charged stimuli and depression.^34,35^ However, we controlled for the role of depression when assessing the relationship between resting α power and trait impulsivity and showed that the relationship was independent from the severity of depression. We also controlled for total LEDD and LEDD dopamine-agonists considering that dopaminergic therapy can enhance impulsivity, and controlled for the severity of dyskinesia as previous studies found an association between dyskinesia and increased α-θ power in the *on* medication condition.^8,36^ We showed that the relationship between BIS-11 and α activity remained strong even after controlling for these factors, perhaps because our recordings were only carried out in the *off*-medication condition. However, because dopaminergic therapy has a key role in triggering ICB in PD patients with predisposing factors, future studies with LFP recordings *on* and *off* medication are warranted to further explore the complex relationship between trait impulsivity, ICB, and dopaminergic medication.

Moreover, we acknowledge that our recordings were carried out only in the *off*-medication condition, because dopaminergic therapy has a key role in triggering ICB in PD patients with predisposing factors, future studies with LFP recording *on* and *off* medications are encouraged to further shade light on the complex relationship between trait impulsivity, ICB, and dopaminergic medications.

We normalized the spectra relative to a very broad frequency range spanning 5–395 Hz to minimize the contributions of other physiological or pathological peaks such as those in the β range. In addition, it is possible that our frequency range of interest, 8–13 Hz, was influenced by the tail of any peaks in the low-β power range. On the other hand, this frequency range was objectively defined as that showing significant correlation with impulsivity in a sweep of all frequencies from 5–35 Hz.

Finally, clinical assessments predated LFP recordings, which might have weakened the behavioral correlations and might, together with the use of the total UPDRS III score, help explain the LFP correlations with motor impairment compared to those previously reported.^37,38^

In conclusion, we show a positive correlation between α power and trait impulsivity in PD patients with and without ICB. Our findings motivate further investigation of α power in PD and other conditions characterized by impulsivity and suggest that this spectral feature might serve as a neural biomarker that relates to impulsive behavior.

## Supplementary Material

Supplementary information

## Figures and Tables

**Table 1:** Demographical and clinical features of the study cohort

ID	Age (y)	Disease duration (y)	Type of electrode	Most affected side	Total LEDD (mg)	D-ag LEDD (mg)	UPDRS-III *off*	UPDRS-III *on*	ICB state	Type of active ICB
01	60	8	Medtronic 3389	L	970	320	28	8	Active ICB	Compulsive shopping, hypersexuality
02	60	15	DB-2202	R	1563	315	50	30	Past ICB	
03	60	6	Medtronic 3389	L	800	0	48	14	Past ICB	
04	60	13	DB-2202	L	1403	280	77	27	past ICB	
05	47	16	DB-2202	R	865	0	71	37	past ICB	
06	53	7	DB-2202	L	635	160	38	25	Active ICB	Hypersexuality, compulsive eating, punding
07	55	16	Medtronic 3389	R	1810	360	51	19	Never ICB	
08	65	15	Medtronic 3389	L	666	0	57	34	Never ICB	
09	52	7	DB-2202	L	1152	180	18	1	Active ICB	Hypersexuality, compulsive shopping
10	66	9	DB-2202	R	1124	360	52	30	Never ICB	
11	65	5	Medtronic 3389	R	995	320	34	16	Never ICB	
12	48	7	DB-2202	R	1180	0	45	34	Active ICB	Pathological gambling
13	61	10	DB-2202	R	500	0	33	12	Past ICB	
14	51	12	DB-2202	L	1020	320	27	13	Active ICB	Hypersexuality
15	56	6	DB-2202	R	1380	480	48	19	Past ICB	
16	66	6	Bsci octode	L	580	0	17	11	Never ICB	
17	67	7	AB-6170	L	820	320	17	5	Never ICB	
18	62	6	DB-2202	R	1082	300	42	13	Never ICB	
19	64	12	DB-2202	L	160	160	52	21	Active ICB	Compulsive shopping
20	63	10	DB-2202	R	780	80	40	17	Never ICB	
21	63	10	AB-6170	R	1800	375	41	11	Past ICB	
22	52	11	DB-2202	L	1470	160	54	27	Never ICB	
23	63	10	DB-2202	R	1275	300	29	10	Never ICB	

Abbreviations: D-Ag, dopamine-agonists; F, female; ICB, impulsive compulsive behavior disorders; L, left; LEDD, levodopa equivalent daily dose; M, male; R, right; y, years; UPDRS-III, Unified Parkinson’s disease rating scale; Implanted leads, Medtronic 3389, (Medtronic, Minneapolis, USA) four 0.5-mm spaced contacts of 1.5-mm length with platinum-iridium cylindrical surfaces; DB-2202, directional leads from Boston Scientific with three segmented contacts on the middle levels (Boston Scientific, USA); Bsci octode, octopolar non directional leads from Boston Scientific; AB-6170, directional leads from St. Jude Medical with three segmented contacts on the middle levels (model 6170, Abbott Neuromodulation, Austin, TX, USA).

**Table 2:** Comparison between PD ICB active and PD-no-active ICB for demographic and clinical data

	PD noICB	PD ICB	P-value
Age (y)	60.6 ± 5.4	54.6 ± 6.0	0.05
Disease duration (y)	10.0 ± 3.8	8.8 ± 2.5	0.7
Total LEDD (mg)	1112.5 ± 410.8	852.8 ± 391.3	0.3
Dopamine-agonists LEDD (mg)	214.7 ± 166.0	190 ± 119.8	0.7
UPDRS-III *off* med	44.7 ± 16.1	34.6 ± 12.6	0.1
UPDRS-III *on* med	19.5 ± 9.4	17 ± 12.0	0.6
UPDRS-IV total	5.5 ± 2.0	4.8 ± 3.3	0.3
UPDRS-IV dyskinesia sub-score	1.6 ± 1.4	1.8 ± 2.2	0.8
RDRS	3.4 ± 2.4	4.4 ± 4.3	0.8
QUIP-RS total score	11.3 ± 15.0	37 ± 17.4	^a^0.003
QUIP-RS ICD score	5.8 ± 7.8	20.1 ± 8.6	^a^0.004
BIS-11 total score	57.1 ± 10.3	63.3 ± 10.1	0.2
BIS-11 attention	13.2 ± 3.1	17 ± 3.8	^a^0.04
BIS-11 motor	21.2 ± 4.1	22.5 ± 1.9	0.3
BIS-11 nonplanning	22.6 ± 6.9	23.8 ± 5.3	0.5
HDRS	6.0 ± 5.1	6.5 ± 6.2	0.8

Values are means ± SD.

a Significant at *P* < 0.05 (uncorrected for multiple comparisons).

Abbreviations: BIS-11, Barratt impulsivity scale; F, female; ICB, impulsive compulsive behavior disorders; ICD, impulse control disorders; HDRS, Hamilton Depression Rating Scale; LEDD, levodopa equivalent daily dose; M, male; QUIP-RS, Questionnaire for Impulsive-Compulsive Disorders in PD-Rating Scale; RDRS, Rush dyskinesia rating scale; UPDRS-III, Unified Parkinson’s disease rating scale-motor part; UPDRS-IV, UPDRS complications of therapy part; UPDRS-IV dyskinesia sub-score, sum of items 32–35.
