# Supplementary material for: Neurophysiological Correlates of Trait Impulsivity in Parkinson’s Disease
Source: Mov Disord. Author manuscript; Available in PMC 2021 Sep 21. (PMC7611688; doi:10.1002/mds.28625)
Supplement: Supplementary information [file EMS128788-supplement-Supplementary_information.docx]

**Supplementary Material**

**NEUROPHYSIOLOGICAL CORRELATES OF TRAIT IMPULSIVITY IN PARKINSON’S DISEASE**

Lucia Ricciardi, Petra Fischer, Abteen Mostofi, Gerd Tinkhauser, Flavie Torrecillos, Fahd Baig, Mark J. Edwards, Erlick A.C. Pereira, Francesca Morgante, and Peter Brown

***Supplementary Material, Methods, Statistical analysis***

***Supplementary Results***

***Supplementary Figure***

***Supplementary Table***

***Supplementary Material***

***Methods***

*Inclusion criteria*

Inclusion criteria for DBS were: a diagnosis of idiopathic PD; age < 70 years; motor fluctuations and L-dopa induced dyskinesias, despite optimal pharmacological management; absence of dementia, major depression with suicidal thoughts or acute psychosis; significant clinical response to levodopa challenge (at least 30% improvement in UPDRS-III score); a disease duration > 5 years. Cognitive impairment was excluded prior to DBS by means of neuropsychological assessment.

*Neurophysiological data analysis*

To assess how many hemispheres displayed a distinct power peak in the alpha (8-13 Hz) and beta (13-35 Hz) range, we performed the following procedure. First, a line was computed between the first and last power value of the band of interest (i.e. 8 and 13 Hz for the alpha band). Then the line was subtracted from the 8-13 Hz power values (or the 13-35 Hz values for the beta band), and the resulting values were divided by the average of that line and multiplied by 100 to provide the relative size of the peak (= the largest normalized power value) in percent. If the highest power value would be located at the edge of the band (at 8 or 13 Hz) in the unnormalized spectrum, the size of the peak would be 0%. Only if the peak was above 5% did we classify that recording as a recording with a distinct peak.

*Reconstruction of contact locations and power distribution*

Assessment of contact localization was made through co-registration of immediate post-operative CT with pre-operative MRI by two experienced neurosurgeons (A.M, E.A.P) specializing in DBS. Assessment was blinded to the electrophysiological data and made using Renishaw Neuroinspire v6 software.

For each participant, a post-operative volumetric CT scan was fused with a pre-operative planning MR scan using custom DBS planning software (NeuroInspire, Renishaw, Stonehouse, UK) to visualise the location of the electrodes relative to brain structures visible on MR imaging. The anterior and posterior commissures were identified. The intercommissural plane was used to define a Cartesian co-ordinate frame of reference with its origin at the midcommissural point (x, lateral; y, anterior; z, superior). The tips and trajectories of the implanted electrodes were defined in this Cartesian space. The point location of each ring contact was defined as its geometric centroid the co-ordinates of which were interpolated based on its known distance from the electrode tip. To establish STN location in co-ordinate space, the axial section with the largest red nucleus diameter on high-resolution T2-weighted MR imaging was determined. In this axial plane, the anteromedial (or ‘anterior’) and posterolateral (or ‘posterior’) poles of the biconvex STN are identifiable. Co-ordinates of the anterior and posterior poles were determined and the centroid of the STN defined as the midpoint between them.

We estimated the alpha power distribution in a space normalized relative to the STN centroid and scaled by the size of each STN by defining the STN anterior and posterior poles as x = -0.5, y = 0.5 and x = 0.5, y = -0.5. The location of each bipolar signal was represented by the average position of the two neighbouring contacts that were used to calculate the bipolar signal, although this tends to shift the power distribution up the electrode track (for example power will increase when contact 0 enters the STN, but this power increase will be attributed to a point 1mm more rostral). To visualize the distribution of alpha power of all 138 bipolar signals (23 patients * 2 hemispheres * 3 bipolar signals) we used the MATLAB function scatteredInterpolant (interpolation method = linear) to interpolate the power values between the contact locations within a 3d meshgrid (another MATLAB function). Note that the edges of the alpha power distribution are less reliable as data points are sparser than within the distribution where there is a higher density of contacts.

*Statistical analysis*

Correction for multiple comparisons was achieved using a cluster-based permutation procedure, which involved the following steps: 1) The GLM was computed on the original data resulting in 30 t-statistics and p-values (one per frequency) for each predictor (QUIP-RS, BIS-11 and UPDRS). 2) A permutation distribution was generated by randomly permuting the association between the STN power and behavioural measurements 500 times (the power of patient 1 was for example assigned to all test scores of patient 5, and so on). The GLM was computed again on each of the 500 permuted data sets and the t-statistics and p-values for the predictors were saved. 3) Finally, the absolute sum of the t-statistics from clusters containing contiguously significant frequency bins was computed based on the original t-statistics and on all the permutation t-statistics. If several significant clusters existed in one permutation data set, then only the largest sum was stored to serve as a comparison value for all clusters found in the original data, which keeps the overall false positive rate at 0.05 (Maris E, Oostenveld R. Nonparametric statistical testing of EEG- and MEG-data. J Neurosci Methods 2007; 164(1):177-90). If no significant cluster existed, the value zero was stored. The last step to test if the original cluster was significant was to count how many of the permutation cluster sums (constituting our null distribution) were larger than or equal to the original sum, divide by 500 (the total number of permutations) and check if the count was below the alpha level of 5%.

Statistical testing for the power difference between the Active and No Active ICB group was performed similarly: The original difference was compared against the differences resulting from randomly permuting the group labels while keeping the group ratio of 6 to 17 (Active vs. No Active ICB) the same.

*Checking the model assumptions*

The multivariate normality assumption was confirmed for the GLM with 8-13 Hz alpha power as criterion by visually examining if the residuals were normally distributed. Additionally, the homoscedasticity assumption was confirmed by plotting the standardized residuals against the predicted values

***Supplementary Results***

*Clinical assessment data*

To evaluate ICB as a trait, we divided patients into 3 groups: PD who have never experienced ICB (Never ICB), PD who had ICB in the past which were remitted at the time of the study (Past ICB) and PD who had ICB at the time of the study (Active ICB). We explored differences in demographic and clinical data among groups (Supplementary Table 1) and found that there was a significant difference in age: The Active ICB and Past ICB groups were significantly younger than the Never ICB group (p=0.016 and 0.043 respectively), but they did not differ from each other (p=0.4). Groups also differed in terms of QUIP total score and QUIP ICD score, as expected, but there was no difference in BIS-11 score or other clinical variables (see Supplementary Table 1). Spearman correlation analysis showed no significant correlation between BIS-11 and total LEDD or LEDD dopamine-agonists (total LEDD: rho=0.34, p=0.109; LEDD dopamine-agonists: rho=0.10, p=0.498).

*Neurophysiological data*

We examined if a distinct alpha power peak was present in all patients. While in nearly all hemispheres at least one contact recorded a pronounced beta peak (41 of 46 hemispheres; screening 13-35 Hz), a distinct alpha peak was recorded in only 25 hemispheres (screening 8-13 Hz).

Our main results were based on LFP power averaged across the bipolar contact pairs of a given electrode and averaged across hemispheres to avoid any channel selection bias and inflation of the sample size. However, to check for laterality effects we determined the average normalized LFP power over 8-13 Hz from the contact pair per electrode that had the highest alpha frequency band power and correlated the result with the BIS-11 score. Significant correlations were present for both hemispheres (Supplementary Fig. 2, Left side: Spearman’s rho=0.61, p=0.002, Right side: Spearman’s rho=0.45, p=0.029), but there was no significant difference in the Fisher’s z-transformed correlation coefficients between the two sides (p=0.484). We also performed correlations with alpha power averaged across the three bipolar signals per side. Only the correlation for the left side was significant, however a direct comparison between the two sides again showed no significant difference (Left side: Spearman’s rho=0.54, p=0.009, Right side: Spearman’s rho=0.35, p=0.106, Difference Left-Right p=0.479).

**Supplementary Figure**

**
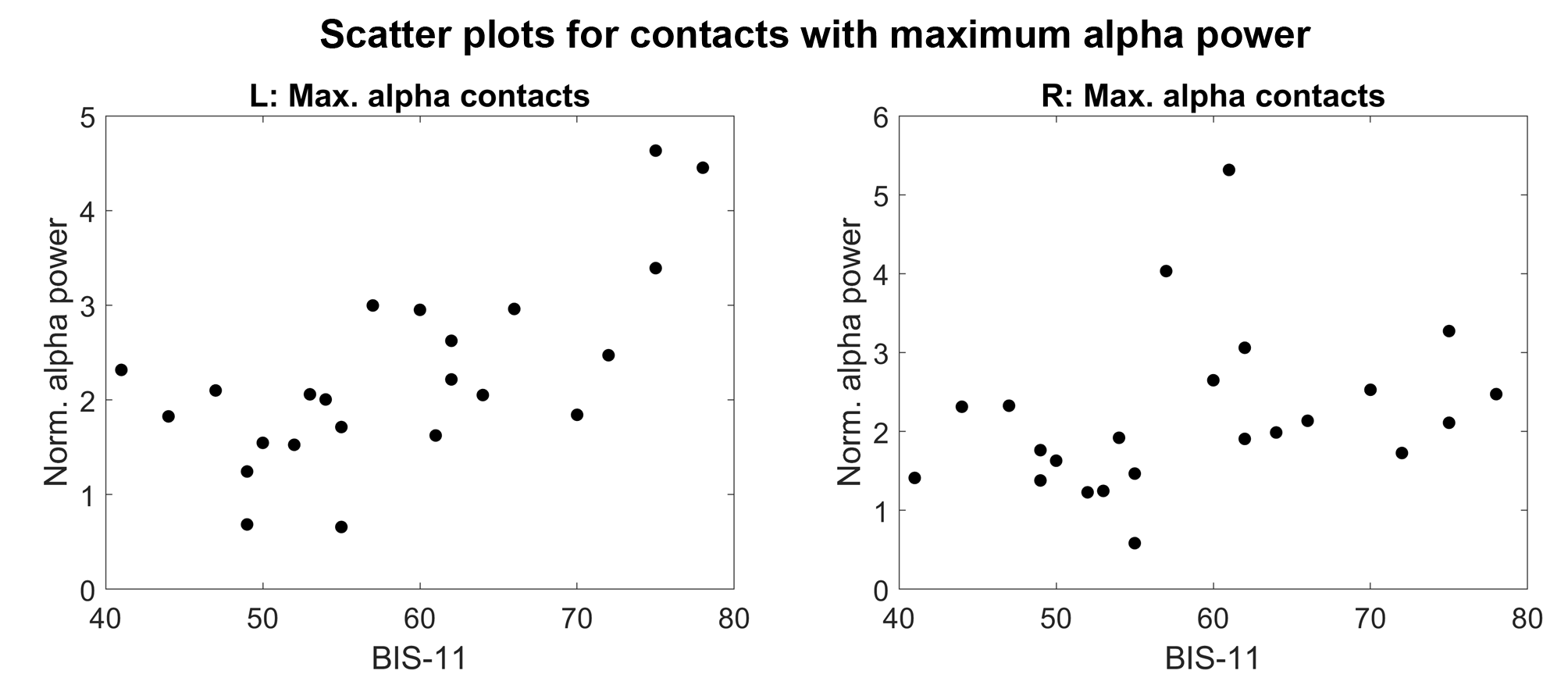
**

**Figure legend:**  Scatter plots of BIS-11 scores and alpha power from the bipolar signals with the highest power. The left and right side show the contacts recorded in the left and right hemisphere, respectively (n = 23).

**Supplementary Table 1: Comparison between PD ICB active, PD ICB past and PD ICB Never for demographic and clinical data.**

|  | PD Never ICB  (N=10) | PD Active ICB (N=6) | PD Past ICB  (N=7) | p-value |
| --- | --- | --- | --- | --- |
| ***Age (y)*** | ***62.4±4.9*** | ***54.6±6.0*** | ***58.1±5.3*** | ***0.024^#^*** |
| **Disease duration (y)** | 9.5±3.7 | 8.8±2.4 | 10.8±4.0 | 0.721 |
| **Total LEDD (mg)** | 1060.2±381.1 | 852.8±391.3 | 1187.2±470.1 | 0.517 |
| **Dopamine-agonists LEDD (mg)** | 220±146.0 | 190±119.8 | 207.1±203.4 | 0.922 |
| **UPDRS-III OFF med** | 39.3±14.7 | 34.6±12.6 | 52.5±15.8 | 0.200 |
| **UPDRS-III ON med** | 18.2±9.3 | 17±12.0 | 21.4±10.0 | 0.711 |
| **UPDRS-IV total** | 4.4±2.3 | 4.8±3.3 | 6.4±2.2 | 0.231 |
| **UPDRS-IV dyskinesia sub-score** | 1.3±0.8 | 1.8±2.2 | 2.0±2.0 | 0.937 |
| **RDRS** | 2.5±2.0 | 3.6±4.2 | 4.1±2.7 | 0.481 |
| ***QUIP-RS total Score*** | ***5.2±5.5*** | ***37±17.3*** | ***20.1±20.1*** | ***0.001^#^*** |
| ***QUIP-RS ICD score*** | ***2.4±2.2*** | ***20.1±8.5*** | ***10.8±10.2*** | ***0.001^#^*** |
| **BIS-11 total score** | 53.3±7.3 | 63.3±10.1 | 62.5±11.9 | 0.127 |
| **BIS-11 Attention** | 12.8±3.1 | 17±3.8 | 13.8±3.1 | 0.104 |
| **BIS-11 MOTOR** | 20.2±4.4 | 22.5±1.8 | 22.71±3.2 | 0.174 |
| **BIS-11 NONPLANNING** | 20.3±6.4 | 23.8±5.2 | 26±6.6 | 0.187 |
| **HDRS** | 5.75±4.5 | 6.5±6.2 | 6.4±6.1 | 0.937 |

Values are mean ± standard deviation. Abbreviations: BIS-11: Barratt impulsivity scale; F: female; ICB: impulsive compulsive behavior disorders; ICD: impulse control disorders; HDRS: Hamilton Depression Rating Scale; LEDD: levodopa equivalent daily dose; M: male; QUIP-RS: Questionnaire for Impulsive-Compulsive Disorders in PD–Rating Scale; RDRS: Rush dyskinesia rating scale UPDRS-III: Unified Parkinson’s disease rating scale-motor part; UPDRS-IV: UPDRS complications of therapy part; UPDRS-IV dyskinesia sub-score: sum of items 32-35. Bold and italic values: significant at p<0.05.

^#^Post hoc analysis: Age: Active ICB vs. Never ICB: p=0.016; Active ICB vs. Past ICB: p=0.366; Never ICB vs. Past ICB: p=0.043.
QUIP-RS total score: Active ICB vs. PD Never ICB: p=<0.0001; Active ICB vs. Past ICB: p=0.101; Never ICB vs. Past ICB: p=0.019.
QUIP-RS ICD score: Active ICB vs. Never ICB: p=<0.0001; Active ICB vs. Past ICB: p=0.138; Never ICB vs. Past ICB: p=0.010.
